# Semiconductor core fibres: materials science in a bottle

**DOI:** 10.1038/s41467-021-24135-3

**Published:** 2021-06-28

**Authors:** Ursula J. Gibson, Lei Wei, John Ballato

**Affiliations:** 1 Department of Physics, Norwegian University of Science and Technology, Trondheim, NO Norway; 2 Department of Applied Physics, Royal Institute of Technology, KTH, Stockholm, Sweden; 3 School of Electrical and Electronic Engineering, Nanyang Technological University, Singapore, Singapore; 4 Department of Materials Science and Engineering, Clemson University, Clemson, SC USA

**Keywords:** Materials for optics, Fibre optics and optical communications, Optical materials and structures

## Abstract

Novel core fibers have a wide range of applications in optics, as sources, detectors and nonlinear response media. Optoelectronic, and even electronic device applications are now possible, due to the introduction of methods for drawing fibres with a semiconductor core. This review examines progress in the development of glass-clad, crystalline core fibres, with an emphasis on semiconducting cores. The underlying materials science and the importance of post-processing techniques for recrystallization and purification are examined, with achievements and future prospects tied to the phase diagrams of the core materials.

## Abstract (web summary)

The application space for optical fibers is growing, enabled by fibers built using special materials and processes. In this Review, the authors discuss the materials science behind producing crystalline core fibers for diverse applications and progress in the field.

## Introduction

Silica glass optical fibres are ubiquitous, with their high transparency and design flexibility enabling the high speed and reliability of modern communications. These attributes of silica-based glasses have driven many complementary applications.

Recently, the use of silica in some specialty applications has been challenged by so-called ‘multimaterial’ or ‘hybrid’ fibres, in which disparate families of materials are co-processed into the same fibre^1–5^. Whereas conventional fibres possess a core and cladding that are compositionally similar (e.g., silica cladding and doped silica core), the multimaterial fibres more fully utilise the stunning breadth of the periodic table. With a broader range of core and cladding materials comes a wider variety of interesting and useful properties. Notable examples include polymer/chalcogenide glass ‘omniguide fibres’^6^ and YAG-derived glass laser fibres^7^.

One fibre material pairing that is under active development is that of a glass cladding and a crystalline semiconductor core. This new type of core opens the door to electronic, thermoelectric, optoelectronic, optically nonlinear, and even mechanical properties not present in glasses^8–11^. The majority of these fibres are fabricated using the molten core draw method (MCD), historically called the molten core method (MCM), where the core phase is a fluid melt that is contained by the glass cladding, with the combination drawn to fibre dimensions. Both unary and compound semiconductors have and continue to be studied with fabrication lengths now routinely well over hundreds of metres, typically limited by preform dimensions. The as-drawn fibres are polycrystalline, which makes them inferior for many applications. A primary consideration is that crystalline disorder, and impurity segregation at the grain boundaries causes optical and electronic defects; scattering and absorptive losses, mechanical weakness and short electronic carrier lifetimes.

The presence of grain boundaries implies multiple nucleation sites during crystallisation, resulting in, especially for alloy systems, inhomogeneity of the core composition due to component segregation at the solidification boundary. Post-processing treatments to promote directional recrystallisation of the core have received increasing attention and have proven invaluable in improving performance. The fibre geometry permits imposition of unusually large spatial temperature gradients and high solidification velocities, in a containment vessel that limits contamination. Fabrication of single crystal cores with aspect ratios of more than 10^4^ is becoming commonplace, and methods for characterising macroscopic samples have been reported^12^, but generally applicable crystallisation methodologies are still under development.

Important advantages of the fibre format include: direct accessibility of the semiconductor phase to the light propagating in the fibre, high surface quality of the semiconductor-in-glass waveguides, internal microstructuring capabilities using thermal treatment after fabrication and the wide choice of core materials/phases. Perhaps less immediate, but nonetheless important is that fibres are a route to single crystal production with reduced energy consumption and conservation of less earth-abundant materials; i.e., the fibre typically utilises the entire precursor semiconductor charge, unlike wafer production and vapour-based processes that may require reclamation of more than 40% of the already purified starting material. Additional interest arises in the assembly of arrays for detectors, where the glass cladding can isolate individual elements, provide mechanical stability and serve as a support network for deposited electrodes. Last, but not least, there are applications that will benefit from the extraordinary thermal and mechanical properties of single crystals with the extreme aspect ratios that are being realised with fibre technology.

This paper connects underlying materials science fundamentals with recent progress in crystalline core fibre processing that has resulted in new structures and materials, and provides a perspective on the past, present and future potential of semiconductor core fibres. It focuses on condensed phase production, concentrating on inorganic fibres made using the molten core technique. High pressure chemical vapour deposition (HPCVD) fabrication is not covered here, although some fibres made using the technique^13^ are included for comparison, when relevant to the discussion. An understanding of the connection between bulk crystal growth and crystallisation within the core during fibre drawing and post-processing underlies the remarkable improvements that have been achieved with laser, plasma and conventional heat treatment of fibres, and is essential to future development in the field.

## History and Background

The earliest glass-clad crystalline core fibres were the metal microwires, drawn within glass cladding, by Taylor^14^. This technique was expanded to include formation of alloy wires, particularly for use as composite reinforcement filaments^15–17^. The possibility of drawing superconducting^18^ and magnetic^19^ wires and the advantages of annealing within the glass cladding^20^ were subsequently recognised. Laser tapering of the fibre has been used to form high quality nanowires of noble metals^21^, anticipating some of the more recent work on post processing of semiconductor core fibres. Beyond the challenges inherent in the MCD method^4^, the importance of crystalline order, the reactive nature of many core elements, and the expansion of the core upon solidification^22^ must be taken into consideration in fabrication of semiconductor core microwire/fibre. These factors are driving research on core recrystallisation schemes. Although glass fibre drawing is usually considered a far-from-equilibrium process, and with the large surface to volume ratio of the fibres the thermal kinetics can be fast, in most cases observed core phases are still governed by bulk thermodynamics. The large surface to volume ratio of the fibre core makes the dominance of heterogeneous nucleation during the draw likely in all but the largest fibres, making the cladding interface quality a critical factor.

**Fig. 1:**
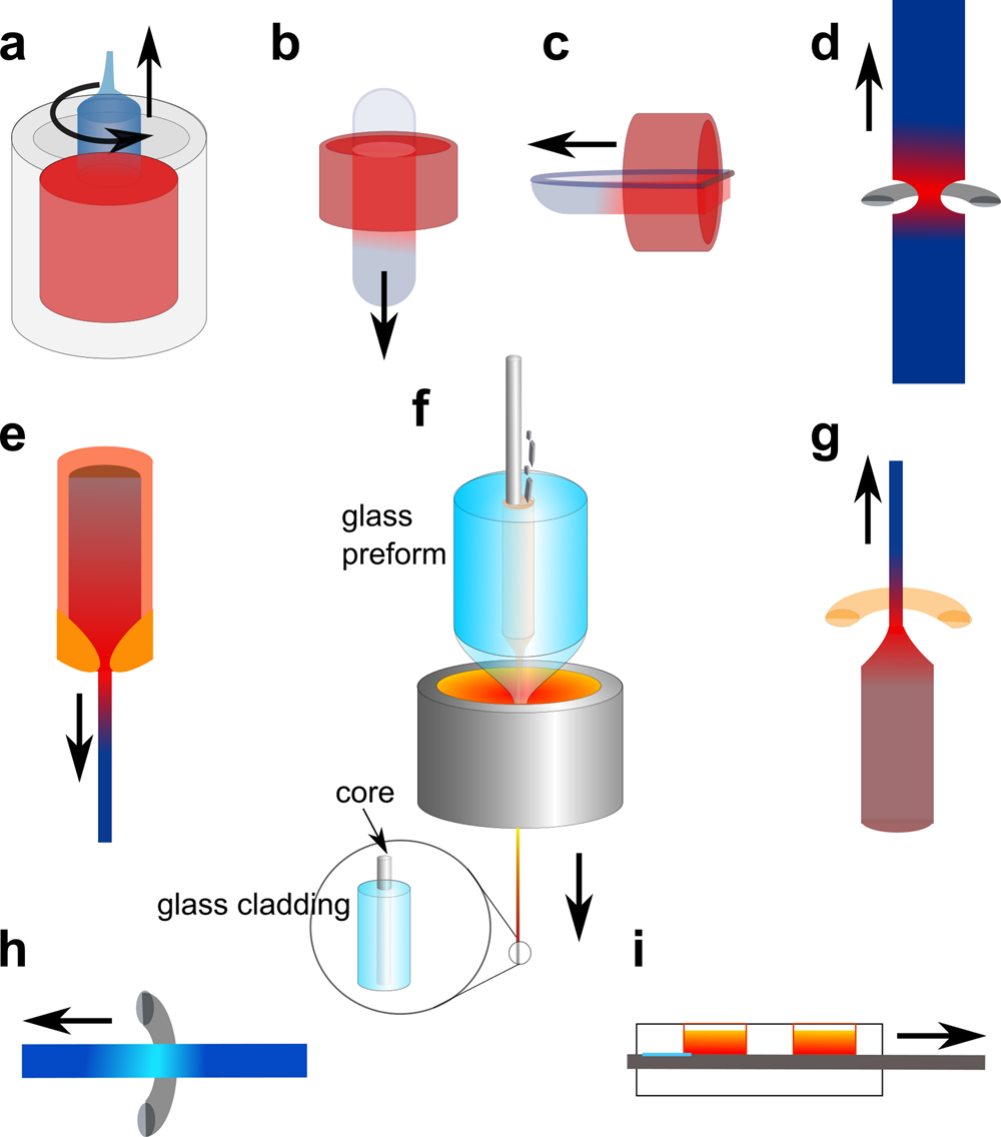
Growth techniques for semiconductors and semiconductor core fibres. Geometries for **a** Czochralski boule growth **b** Bridgman vertical growth, **c** Bridgman horizontal growth, **d** float zone growth (optical and RF heating possible), **e** micro-pulldown method, **f** molten core fibre drawing (adapted from^46^), **g** laser heated pedestal growth, **h** traveling solvent float zone method, and **i** liquid phase epitaxy. In all these techniques, a moving thermal gradient allows solidification, and in some cases, diameter changes during the crystallization of the semiconductor.

It is informative to review growth techniques for bulk semiconductors as shown in Fig. 1 before discussing the MCD production and recrystallisation of semiconductor core fibres illustrated in Fig. 1f. Most available bulk (boule) growth information is for silicon, due to its technological importance, with Czochralski^23^, Bridgeman^24,25^ and float zone (FZ)^26^ methods prevalent, and both the scientific and patent literature are valuable background resources. Liquid phase epitaxy (LPE) was developed for growth of layered structures in semiconductors, often at temperatures below the bulk melting point of the seed wafer. Although this technique has been superseded by vapour-based methods, the LPE literature is relevant to the processes that occur during melting and recrystallisation of compound or alloy semiconductor core fibres.

For each of the techniques, factors that aid in the understanding of the molten core drawing of semiconductor core fibres or their post-processing are highlighted. Key considerations are the thermal gradients that are maintained, the sources of (impurity) oxygen/oxide incorporation, crystal interface growth velocity, residual stress, surface tension, high thermal conductivity of the semiconductor core melt, fluid flow in the core and the interaction between the core and cladding. While not all of these are reported for each fibre developed, the community is moving towards an awareness of their importance, and detailed information is increasingly available.

### Czochralski (CZ) growth

As illustrated in Fig. 1a, CZ growth^23^ is the most common method used for the production of silicon crystals. A crucible (typically silica), with or without treated surfaces, is used to contain molten silicon, and a rotating crystal seed is contacted to the surface of the melt, and withdrawn slowly, permitting attachment of atoms from the melt to the seed, which is at a slightly lower temperature. Control variables include thermal gradients, crucible coatings and the choice of seed crystalline orientation. Huang, et al.^27^ characterised the temperature gradients present during growth of 70 mm diameter boules, and found maximum values that ranged between 30 and 60 K cm^−1^ near the growth interface, almost surely less than that experienced by a fibre as it exits the draw tower furnace.

An inert gas atmosphere, typically argon, is employed to minimise oxygen uptake, but interaction between the molten silicon and the crucible typically leads to incorporation of oxygen^28^ at a level of 10^18^ cm^−3^. Fibre fabrication is usually performed under inert atmospheres, but one critical difference is the higher temperatures employed for drawing the silica cladding tube into fibre, typically between 1900–2000 °C.

In the pursuit of easier removal of the residual silicon after CZ boule growth, as well as reduction of oxygen contamination, crucible coating materials including Si_3_N_4_ and BaO derived from either the hydroxide^29,30^ or silicate^31^ coatings have been investigated. The patent literature on CZ crucibles is an especially useful source of information for drawing of silicon core fibres when searching for interface/coating material miscible with the silica preform and yielding a suitable viscosity at the fibre drawing temperature yet forming a barrier to oxidation of the core. Although there are no known reports of fibres drawn with this interface coating, zirconium compounds have been investigated for silicon boule growth^32^. The coatings developed for bulk growth are a good starting point, but softening of the bulk crucible is not desired, and complete de-wetting of the inside of the glass preform, while helpful for the formation of semiconductor beads, is not desirable for formation of continuous core fibres. The ideal for the fibre is to form an interfacial bond that suppresses the transport of oxygen to the core and tolerates deformation at the solidification temperature of the core.

### Bridgman–Stockbarger method

Both vertical^24^ (Fig. 1b) and horizontal^25^ (Fig. 1c) directional resolidification techniques are primarily used for compound semiconductor materials, and also use a crucible. This technique relies on a controlled thermal gradient and has permitted the growth of sizable crystals of many high temperature materials. The literature on bulk crystal growth highlights the importance of the temperature gradient, which has been related to the structure of fibre cores, both for the drawing process and in post fabrication anneals. In fibres, there has been little explicit investigation of the temperature gradient during fabrication, as the temperature field is not easily measured, and is typically fixed for the particular draw tower furnace employed. Varying the draw speed can yield some information, but the furnace temperature typically has to be adjusted for successful draws at different speeds, complicating analysis of the temperature gradient. Background information from the Bridgman literature can often be scaled to predict the influence of the gradient on fibre production and recrystallisation. Some information is available on temperature gradients and crystallisation front speeds during fibre post-processing, and recognition of this as an important control variable is increasing.

### Float zone (FZ) growth

This process, shown in Fig. 1d, is worthy of attention as it resembles the recrystallisation process used for fibre cores, with the differences that for the bulk technique there is no crucible, reducing stress effects and that convection forces must typically be considered. Stresses typically differ substantially for pure and alloy semiconductors^33^ where variations in the surface tension as a function of composition complicate analysis.

For FZ growth, an inert gas or vacuum environment is used, and surface tension of the silicon allows the translation of a melt zone vertically along the length of a high purity rod. For RF induction heated furnaces, convection is reduced, magnetic forces stabilise the melt and temperature gradients^34–36^ can be tens of K cm^−1^. The stability of the melt zone limits the diameters that can be grown as well as the uniformity of the cross-section. Although for fibres, the cladding can reduce these considerations, if the processing temperatures are too far above the softening temperature of the glass, stability will be compromised. Typical bulk growth rates are on the order of mm min^−1^ and the largest FZ temperature gradients are on the order of 500–1500 K cm^−1^ in optical furnaces^37–39^ during oxide crystal growth, comparable to fibre recrystallisation conditions. CO_2_ lasers can also be used as the heat source for FZ growth^40^. For FZ silicon boules, carbon and oxygen residuals are extremely low (on the order of 10^16^ cm^−3^), but the presence of the oxide cladding in the case of fibres may not permit such low impurity levels. However, segregation coefficients are a function of both temperature gradients and crystal growth rate—two variables that can be controlled over a wide range in fibre core recrystallisation, suggesting that appropriate conditions may yield major improvements.

### Micro-pulldown method

The micro-pulldown method illustrated in Fig. 1e, where the melt is suspended above the seed, and a crucible nozzle controls the diameter of the melt cylinder, is also of relevance to semiconductor fibre fabrication. High temperature polycrystalline metallic alloys of mm diameter have been successfully drawn at rates of up to 200 mm min^−1 ^^41^. Silicon is a challenging material to process with this technique, due to the high surface energy and low viscosity of the liquid, but the technique has been used to fabricate bare single crystal silicon fibres with diameters of 0.2 to 1.0 mm^42^, with growth rates of 0.1 to 5 mm min^−1^. Temperature gradients were reported to be up to 3 K cm^−1^. The viscosity and surface energy considerations again anticipate the utility of an interface modifier for silica-encapsulated Si growth to create a surface that will be wet sufficiently by the silicon to avoid separation from the glass, yet avoid the formation of spherical inclusions during or after the draw process^43,44^.

### Laser heated pedestal growth (LHPG)

Although its primary application has been in the growth of oxide fibres^45^, LHPG studies using the geometry of Fig. 1g are of relevance to fibres because the thermal gradients and the optical systems used for feed-rod heating presage much of the laser treatment of fibres. While the unconstrained surface may lead to unwanted diameter and surface quality variations, temperature gradients with the laser pedestal growth technique can reach 10^4^ K cm^−1^, comparable to the values reported for CO_2_ laser treatment of fibres^46–48^. More refined versions of this optical technology are used for the fabrication of the quartz suspension fibres used for gravity wave detection^49,50^, and are now commercially available for tapering of optical fibres (e.g. Nyfors^®^, Fibrebridge^®^). Tapering stations have been successfully used in the recrystallisation of silicon core fibres^51,52^, resulting in high optical quality crystalline cores.

### Travelling solvent FZ

The travelling solvent FZ method^53^ utilises a (typically low solid solubility) addition to the melt to promote formation of a liquid region at temperature lower than the melting point of the (pure) semiconductor of interest. This melt zone can be translated through the boule as shown in Fig. 1h, leading to controlled recrystallisation. In bulk, this has been used to produce mm scale SiGe rods with an estimated temperature gradient of 0.65 K cm^−1^, and a growth rate of 0.16 mm min^−1^. Ge was a solvent for the alloy^46^, suppressing nucleation due to the lower melting point of the Ge-enriched material near the solidification front. With the use of a seed crystal, single crystal material with a diameter of 8 mm could be grown^54^. The recrystallisation of SiGe core fibres and silicon in the pseudo-eutectic Si/GaSb are other examples of solvent-assisted formation of a crystalline core^55^, and the literature on this method is a valuable resource for new concepts in processing of semiconductor core fibres.

### Liquid phase epitaxy (LPE)

In addition to literature on the travelling solvent method, that on LPE, shown in Fig. 1i, is of key importance in systems where disproportionation of the initial semiconductor charge takes place, e.g. the drawing of InGaSb core fibres^56^. In this system, metallic Sb filaments were observed in the as-drawn fibres, and this excess metal served as a solvent for recrystallisation of the ternary alloy^12^. Intentional introduction of an excess of one component of a binary system (e.g. Ga in GaSb) can be used to establish a crystallisation process akin to LPE but in a closed vessel where oxidation concerns are much reduced. However, crystal growth rates are typically much higher with the fibres than are used in LPE, and limited reports of a gradient perpendicular to the growth front for the latter technique appear in the literature, with maximum values of 25 K cm^−1 ^^57,58^. Despite differences in conditions, the knowledge acquired by the LPE community is applicable to the fibre core and may be useful in the exploration of new materials and combinations of solvents for travelling zone growth.

### Laser drawing/tapering

Lasers are of major interest for recrystallisation of the fibre cores but have other applications as well. In one study^59^, a CO_2_ laser system was used to draw Ge core, silica clad fibre from an 8 mm preform with a 220 µm Ge rod inserted. The success of CO_2_ laser heating in the fabrication of fibres^60^ for the LIGO suspension verifies laser fibre drawing is a promising approach, and there is at least one fibre draw facility with a high-power CO_2_ laser installed^61^. Thermal modelling of the fibre draw is complicated by the necking down of a large preform^62^, but modelling has been performed for CO_2_ laser tapering of silica fibres^63^, and since the core is a small percentage of the cross-section for most semiconductor fibres to date, this analysis is a useful starting point for analysis of laser post-processing.

## Semiconductor core fibres

Some of the earliest semiconductor-core optical fibres were made using HPCVD^64^ inside a pre-drawn micron-size pore of a glass capillary. Advantages of this method include the high purity that can be achieved with gas precursor reactants and the ability to deposit materials that would vaporise in a conventional (MCD) process. The lower temperatures used (well below the melting point of the deposit) result in either amorphous or small-grain polycrystalline materials, which can be useful for some applications. The fibres made by this technique are typically tens of cm long, and involve substantial growth times, due to the challenges of transport through the small pore diameters. Laser annealing of these materials has been used to make single crystal cores.

Spliced fibres with different inner diameters can be used to force a molten core material into a smaller diameter pore, as shown early on with gold cores^65^, and there are now significant numbers of papers that rely on this ‘pressure-assisted melt filling’, (PAMF) particularly for the fabrication of Ge core materials^66,67^.

Fibre drawing using the molten core method (Fig. 1f) has been more widely adopted, using both reduced scale commercial^68^ and laboratory scale^69^ towers. For this technique, the (bulk) semiconductor is introduced into a cavity in a glass preform that has a softening temperature above the melting point of the semiconductor. The glass cladding serves as a deformable crucible for the core liquid during glass fibre drawing. The advantages of this technique are the scalable nature, with hundreds of metres of fibre^47,70,71^ from individual production runs, and the ability to explore new materials systems rapidly without equipment modification. A limitation of this technique is that the fibre cannot be drawn with a cladding glass that softens above the sublimation temperature or boiling point of the core constituent(s).

### Post-draw processing methods

**Fig. 2:**
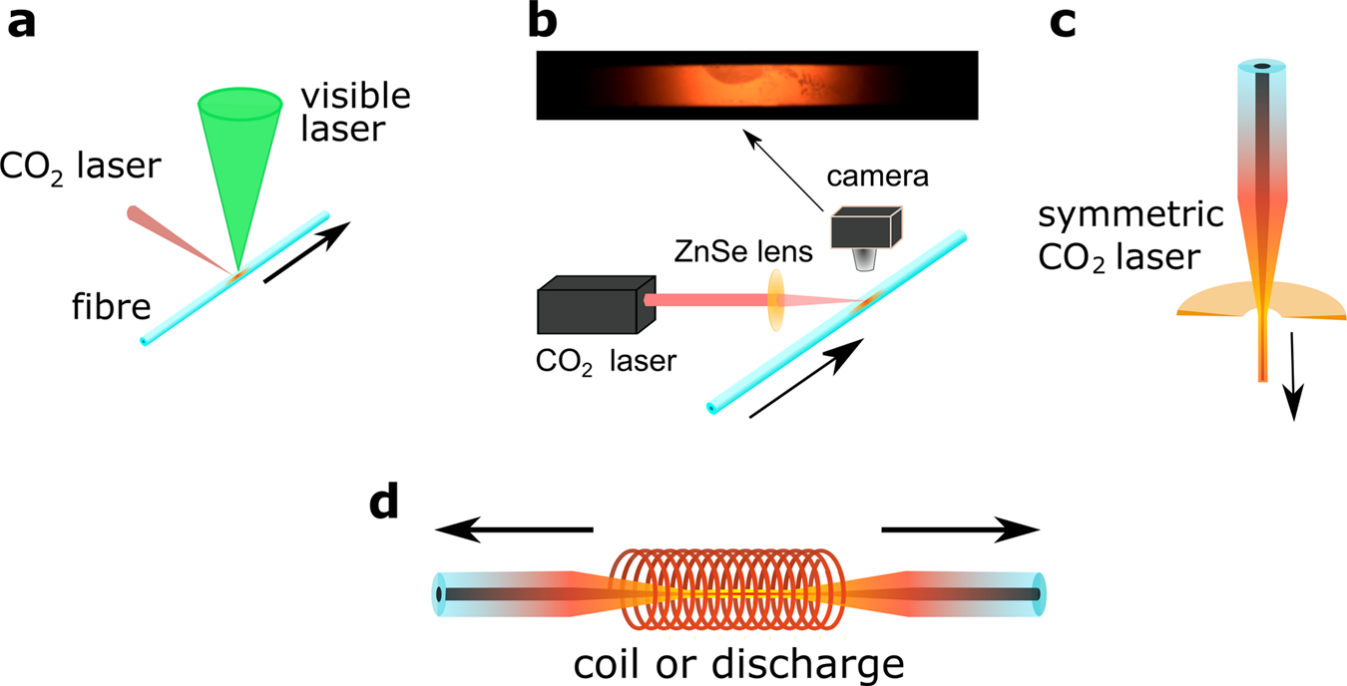
Post-processing treatments for semiconductor core fibres. Thermal gradients are on a smaller length scale than for bulk processing and the thermal transfer is efficient, allowing imposition of cooling rates not possible in bulk growth, particularly with laser heating. **a** Dual wavelength treatment to heat the core directly in addition to cladding thermal transfer, **b** unidirectional CO_2_ laser heating (adapted from [46], **c** symmetric beam CO_2_ laser heating, and **d** use of tapering equipment.

Methods that have been used to perform post-fabrication annealing/recrystallisation of continuous crystalline core fibres include oven thermal annealing^72^, rapid photothermal annealing^73^, laser treatment (at both visible^74^ and IR^46,47^ wavelengths) and diverse heating methods in tapering rigs. The non-standard treatments are illustrated in Fig. 2. Diffusion constants in the solid, and hence oven thermal treatment below the melting temperature of the core is slow. Laser-driven translation of a melt zone through the core has dominated recent reports, because it is rapid, and if the molten zone is long in extent compared to the core diameter, core variations on a mm length scale can be minimised. Among these techniques, the largest reported temperature gradients are with laser treatment^13,75^, while tapering and oven/thermal annealing have lower peak temperatures and spatial gradients. The rapid melt/recrystallisation cycle with the extreme temperature gradients possible (>10^4^ K cm^−1^) can be expected to segregate impurities both by thermomigration and due to liquid-solid segregation coefficients. The surface tension of the liquid semiconductor in the fibre has not been extensively investigated. However its importance should not be minimised, as both flame heating^43^ and CO_2_ laser heating^48,55^ have been used to form encapsulated spheres in silica due to the high surface tension of liquid silicon.

For unary semiconductors, rapid translation of the melt zone can minimise the growth of additional nuclei thereby reducing polycrystallinity effects, while for alloys, finite diffusion coefficients and constitutional undercooling make slower translation preferable. For alloy cores, there will be concentration transients at each end of the treated region, but the length of these regions is generally only a few core diameters. An advantage of multiple component systems is that it is generally easier to produce single crystal material, as the lowest melting point component can act as a travelling solvent.

## Materials and Phase Diagrams

In the selection of a suitable cladding glass, and hence draw temperature and post-processing conditions, the melting (and/or vaporisation) temperature of the semiconductor core must first be considered. If there is more than one component in the core, the nature of the (equilibrium) phase diagram, the compositional breadth of two-phase fields, the presence of a eutectic point or additional phases, etc. should ideally be known. The fibre draw process is rapid enough to suppress macroscopic segregation of phases in most cases, but post-processing imposition of thermal gradients can produce a variety of compositional structures. In the discussion below, the studied fibre systems are divided according to the class of phase diagram of the core. The phase diagrams anticipate the phases realised and structures adopted during fibre drawing and post-processing.

### Single component cores Ge, Si, Se and Te

For the semiconducting elements, the phase information at ambient pressure is limited to the melting and vaporisation temperatures. Unary semiconductor cores realised to date include silicon, germanium, selenium and tellurium. For elemental cores, the primary quality considerations are crystalline grain size, reaction with the cladding glass, stress effects and impurity incorporation, particularly as aggregates at grain boundaries.

Some unary fibres have been used as-drawn, but most devices reported have been based on thermally treated fibres. In at least some of the as-drawn studies, single crystal regions were inadvertently selected after HF removal of the cladding, as the etch attacked grain boundaries^76,77^ and reduced the polycrystalline material to powder. When the cladding is left intact, the core can be heat-treated in situ, and characterised to develop suitable processing conditions for the growth of single crystals. Reduction of grain size is driven by excess nucleation, which can be altered by the temperature/time parameters used during fabrication or recrystallisation, the chemistry of the cladding glass and the choice of an interface coating. In some cases, such as aluminium in silica, the core-cladding interaction is so strong that oxidation of an Al core led to formation of a silicon-core fibre^78^, as anticipated by the Ellingham diagram. Less dramatic interaction may still be sufficient to produce stable inhomogeneities that nucleate new grains. The thermal gradient and translation speed of the crystallisation front, while not always specified, are of importance in the competition between seeded regrowth of a unary core^47^ and nucleation of additional grains.

Germanium was one of the first semiconductor core fibre materials explored^79,80^ (using HPCVD), with seminal papers highlighting the potential for cores with both Ge and additional components. The optical losses of the amorphous as-deposited Ge fibres were reported to be above 15 dB cm^−1^ at a wavelength of 3.5 µm, dropping to 4.8 dB cm^−1^ at 10.6 µm. Subsequent studies on crystallised cores have demonstrated the potential of this material for mid-IR devices^81^.

Partly due to the accessible melting point of Ge (~940 °C) this material has been studied extensively using other fabrication methods. Early work included photonic crystal fibres where molten Ge was forced into hollow capillaries in the structure^66^, resulting in composite fibres with cm-long filled cores, and temperature dependent optical loss, as measured by propagation through the glass. Pressure assisted melt filling was also used to make a hybrid 3–7 mm long fibre section with a Ge core in proximity to a conventional optical fibre core as a photodetector^82^ with a linear response to a wavelength of 1.3 μm. Recent single-core fibre experiments were reported^67^, with pressure assisted melt-filling followed by CO_2_ laser annealing to increase grain size and reduce core stresses via recrystallisation. The laser treated fibres had core diameters of ~2 µm and were single crystals over 8 mm long (limited by the equipment).

MCD draws of Ge core fibres have been made with both borosilicate^70,81,83,84^ and silica^85^ cladding glasses, with the latter having optical losses of 0.7 dB cm^−1^ at 3.39 µm. Ordu, et al.^84^ reported an average optical loss value of 5.1 dB cm^−1^ over the wavelength range 5.82 to 6.28 µm. Borosilicate glass clad Ge with 20 µm core has been fabricated in 100 m quantities using a double-draw method, and demonstrated high speed photodetection^70^. This double draw procedure was associated with a slight reduction in stress, and electrical resistivity was measured to be about twice the value of the starting material, possibly due to cladding interactions.

Post-fabrication processing has been performed on Ge core fibres with tapering and oven annealing as well as argon and CO_2_ laser annealing. The tapering studies^86^ provided the first evidence of preferential crystal orientation after recrystallisation, with single crystal regions up to 4 mm in length. While oven annealing was reported to increase the grain size and reduce stress in Ge core fibre, no functional properties were reported in that study^87^. The low level of interaction between Ge and silica has been exploited in fusion splicer annealing for the formation of microsphere temperature detectors^88^. Laser treatment of HPCVD Ge core, silica-clad fibres^89^ with 488 nm light resulted in a 9 mm single crystal with optical losses of 1.33 dB cm^−1^  measured at a wavelength of 2 µm. CO_2_ laser annealing of a Ge core fibre with an axially symmetric beam^90^ (although the fibre was not on-axis) decreased stress and optical losses, with Raman studies used to explore the degree of interaction between the 30–60 µm core and the silica cladding. A minimum in optical losses was observed as a function of CO_2_ laser power with a scan rate of 3 mm s^−1^, but grain size was not reported.

While Ge has been less studied optically because the small bandgap makes it incompatible with conventional telecom test equipment, losses in the material have been reduced^89^ and nonlinear behaviour has been explored^91^. Whispering gallery modes have been characterised in microspheres made from Ge core fibre^92^. Further interest in the material is expected due to its demonstration as an infra-red Raman source^93^ with a 5.62 µm pump.

Some of the salient results of post-fabrication treatment are summarised in Table 1. Post fabrication laser and oven treatment has been shown to improve the mechanical and optical properties of Ge core fibre, but the available information spans a wide range of conditions, making firm statements about the best path forward challenging.

**Table 1:** Summary of germanium and silicon annealing conditions and results for fibres made by high pressure chemical vapour deposition (HPCVD), pressure-assisted melt filling (PAMF) and molten core drawing (MCD).

	Ref	Fabrication	Core diam	Anneal method	Crystal length	Anneal speed	T gradient
Ge	^80^	HPCVD	5 μm		11 mm		
	^89^	HPCVD	5.6 μm	488 nm Ar laser	9 mm	1 mm s^−1^	
	^67^	PAMF	2 μm	10.6 μm CO_2_ laser	8 mm (stage limit)	2 mm s^−1^	
	^86^	MCD	8 μm	taper	2–4 mm	2 mm s^−1^	600 K cm^−1^
	^90^	MCD	3–60 μm	CO_2_ 3-beam		3 mm s^−1^	
Si	^13^	HPCVD	2–5 μm	488 nm Ar laser	5.1 mm	1 mm s^−1^	4000 K cm^−1^
	^73^	MCD	160 μm	xenon lamp	9 mm	~	
	^47^	MCD	12 μm	10.6 μm CO_2_ laser	2.5 cm (stage limit)	0.1–3 mm s^−1^	12000 K cm^−1^
	^51^	MCD	2 μm	taper	9.2 mm	0.4–0.45 mm s^−1^	

Silicon is the most-studied core material employed to date, due to its transparency at telecom wavelengths and the substantial expertise developed through planar silicon photonics. There are a number of reviews of this core material already^9,51,94–96^, and here, a few additional studies will be highlighted.

Most MCD fabrication of silicon core fibre has been performed with bulk silica as the cladding, and post-processing has led to dramatic improvements in optical losses and the grain size of the core material^47^. High quality fibres were reported in some cases with just the silicon/silica combination, while other studies used an interface layer to reduce stress during drawing of cane and fibre and to lessen oxygen incorporation in the core. The role of stress, due to the Si expansion upon solidification, cannot be ignored, as it is sufficient to limit the maximum core size that can be drawn. Rapid cooling of microspheres formed by local heating of silicon-core fibre^12^ was shown to result in lower tensile stress than either the as-drawn fibre or spheres formed with more gentle temperature cycles. The relaxation of the glass surrounding the silicon is likely responsible for this difference, and choice of glass or interface layer to retain a deformable material during the solidification of the semiconductor can be important; the increase in volume for silicon going from the melt to room temperature is almost ten percent.

To date, there is no definitive study of the role of time/temperature influence on the incorporation of oxygen from the cladding into the molten core, but there is suggestive evidence that the greater thermal mass of a larger preform, requiring longer high-temperature exposure, leads to to more oxygen inclusion in the core. Typically, only the preform size, heater arrangement and possibly the temperature (measured with varying degrees of accuracy) are reported; a meaningful parameter for thermal exposure would require integration over the thermal field and draw time in the furnace. A short heating cycle with a low temperature draw^97^ has been one approach to reduction of optical losses; introduction of an interface layer that acts as an oxygen barrier is another^98^. The latter technique, based on CaO coatings, has been used by the group at NTNU in the production of fibres, in tower^47^, hand drawn^98^, and laser redrawn^55^ fibres with core diameters down to 1.5 µm. While there is some evidence that coating or reactive oxide inclusions^99^ contribute to heterogeneous nucleation in the core, thermal post-processing with thermal gradients promotes migration of these inclusions out of the annealed core, based on optical transmission results. A tapering rig has been used to produce submicron cores of high quality with the interface-coated material^51^.

There are two additional techniques where the thermal mass/density of the cladding was altered for drawing silicon fibres, one where a silica sol-gel was used to support the core, then densified and drawn, and the other where an array of ‘partially evacuated’ hollow tubes^71^ were part of the cladding. The sol-gel cladding report^100^ showed Raman shift differences of ≤0.5 cm^−1^ between the rod used as starting material and the condensed sol-gel encapsulated silicon, with that small shift reverting to the original value in the as-drawn fibre. In the other study^71^, somewhat lower resolution data suggested Raman shifts of 1.5 cm^−1^ from the value for pure silicon. The geometry used for the Raman measurements was not specified, which is of relevance in light of the studies on Ge core fibre^90^, where the Raman shift was observed to vary over the cross-section of the core. For the stacked tube configuration^71^, optical losses below 0.25 dB cm^−1^ were reported for the NIR, and less than 2 dB cm^−1^ for the mid-IR, with transmission ‘up to at least [λ=] 4 µm’.

Post-fabrication processing of silicon fibres has been performed using both commercial fibre processing equipment as well as Ar ion and CO_2_ lasers, as detailed in reviews^9,94–96^ and papers dedicated to the annealing process^13,47,51^, and summarised in Table 1. Although most studies are on continuous fibres, there is also work on the formation of either attached^69,101^ or segregated microparticles and p-n junctions formed between joined microsphere pairs^43^. A recently introduced method for recrystallisation of silicon is the introduction and removal of a solvent metal with a low residual solid solubility. This allowed recrystallisation at reduced temperatures, and resulted in fibres with broadband IR and THz transmission^102^ despite residual solvent impurities.

In addition to improvement of the core material induced by recrystallisation, two key processing techniques are worthy of mention: tapering and splicing. These have been driven by optical applications, where a reduced cross-sectional area of the core increases the optical intensity, and thus non-linear behaviour of the core^51,86,103–106^, and for coupling to conventional silica fibres^97,107–109^. Tapering has been shown to favour formation of single crystal core material, with the thinning of the fibre likely increasing the thermal gradient during solidification^51^.

The large amount of effort that has been devoted to the development of silicon core fibres has moved this material the closest to commercial applications and has informed processing and materials studies for a wide range of additional materials. Although there are several studies that suggest it is possible, pure silicon fibre with a single crystal core of metre length has not yet been reported, but to date, applications have not required such a fibre.

### Other elements: Se, Te

Additional elemental semiconductors have been fabricated in the cores of glass-clad optical fibres, with Se receiving particular attention due to its low melting point (220 °C) which makes it compatible with polymeric claddings^110^. Se core glass-clad fibres^111–113^ have been reported with optical losses as low as 1.5 dB cm^−1^ at a wavelength of 1.3 μm, when a two-stage oven anneal was performed on amorphous-core starting material. Tellurium has also been drawn into glass-clad fibre, but initial experiments showed large optical losses due to incorporation of cladding components in the core^114^. The chalcogenides have higher vapour pressures, and are thus more often drawn in lower temperature claddings such as polymers or borosilicate glasses, where thermally-driven vapour pressure effects are suppressed. Thicker cladding, or spatial distribution of the chalcogenide material before drawing may reduce vapour pressure related problems.

### Binary and ternary systems

The inclusion of additional element(s) in the core can have profound implications for the production of single crystalline fibre cores and their electrical, optical and optoelectronic properties. In addition to the wider range of properties of the solidified core, employing an alloy can permit adoption of lower processing temperature, potentially reducing glass cladding interactions. However, obtaining microscopically homogeneous materials can be challenging, even in the case where equilibrium phase diagrams indicate the existence of solid solutions. Although the fibre geometry allows imposition of large thermal gradients, and rapid cooling during translational annealing/recrystallisation, limitations introduced by finite diffusion velocities remain.

**Fig. 3:**
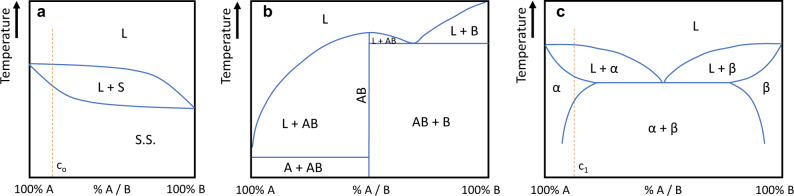
Classes of simple phase diagrams discussed in this paper. Elements may combine to form **a** isomorphic solid solutions, **b** a line compound with additional phases, and **c** eutectic systems, which may have extended solid phase fields (α and β). L liquid, S solid, S.S. solid solution, AB line compound, α A crystal with B substitutions, β B with A, c_n_ concentration under study.

The binary systems studied to date include solid solution isomorphic systems (Fig. 3a), line compound semiconductors (Fig. 3b), and eutectic systems (Fig. 3c), as well as alloys that have many intermediate phases. The discussion here will be organised according to the complexity of the phase diagram. When more complex compositions are considered, two of the elements can form a compound that segregates stoichiometrically, forming a pseudo-eutectic. These are presented in the section on ternary compounds, but the principles of a simple eutectic are applicable. For ternaries of III–V materials, an additional element from one of these groups is often substituted, altering the bandgap, but not the crystal structure.

It is important to recognise that if diffusivity is low, the phase diagram can be misleading, i.e. phases may be sufficiently stable once formed that the composition of a region will not change noticeably after solidification, despite the changes suggested by the equilibrium diagram. This is true both for isomorphic binary systems where the solid has a composition different from the liquid, e.g. for C_0_ in Fig. 3a, and for pro-eutectic solids, particularly where there is a significant compositional extent to the solid solution phase field at the extrema of the eutectic tie line, e.g. such as C_1_ in Fig. 3c and solid α material, once formed, may not revert to a mixture of α and β.

### Binary alloys

When the chemical properties of the constituents are similar, and the atomic radii are not too disparate, isomorphic solid solutions with random substitution of one element for another are possible. Si_x_Ge_(1-x)_ and Cu_x_Ni_(1-x)_ are the classic isomorphs presented in materials science textbooks, as any composition of the alloy is thermodynamically stable, once produced.

#### SeTe

Selenium and tellurium demonstrate this phase behaviour^115^, with Se having the lower melting temperature. Phosphate glass clad fibres with crystalline Se_*x*_Te_*(1-x)*_ cores^116,117^ have been studied, with *x* values equal to 0.5 and 0.8. The as-drawn material was amorphous for both compositions, and post-fabrication annealing at 150–160 °C was used to crystallise the material. These fibres were utilised as stress sensors^117^ and photoconductors^116^.

#### SiGe

**Fig. 4:**
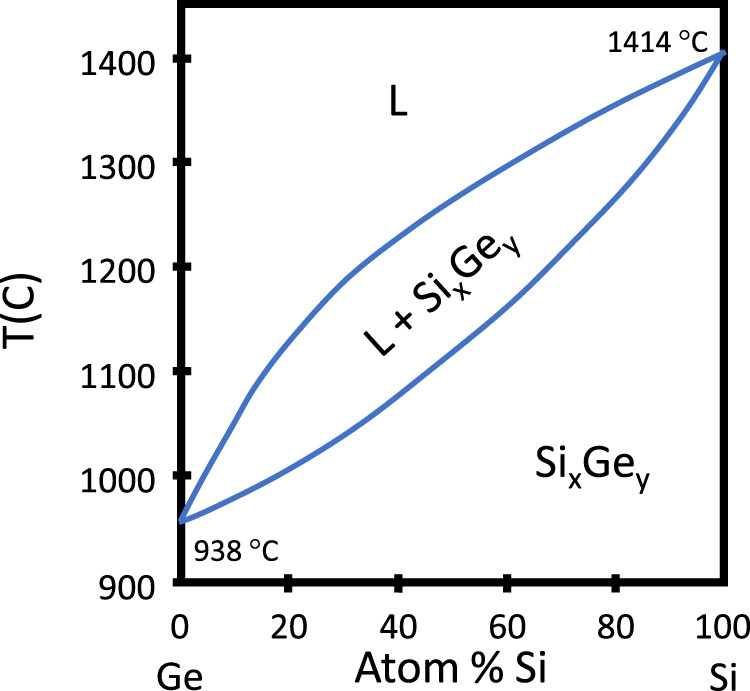
Phase diagram for Si and Ge. While any composition solid solution is possible, kinetics often lead to local inhomogeneities. L – liquid.

**Fig. 5:**
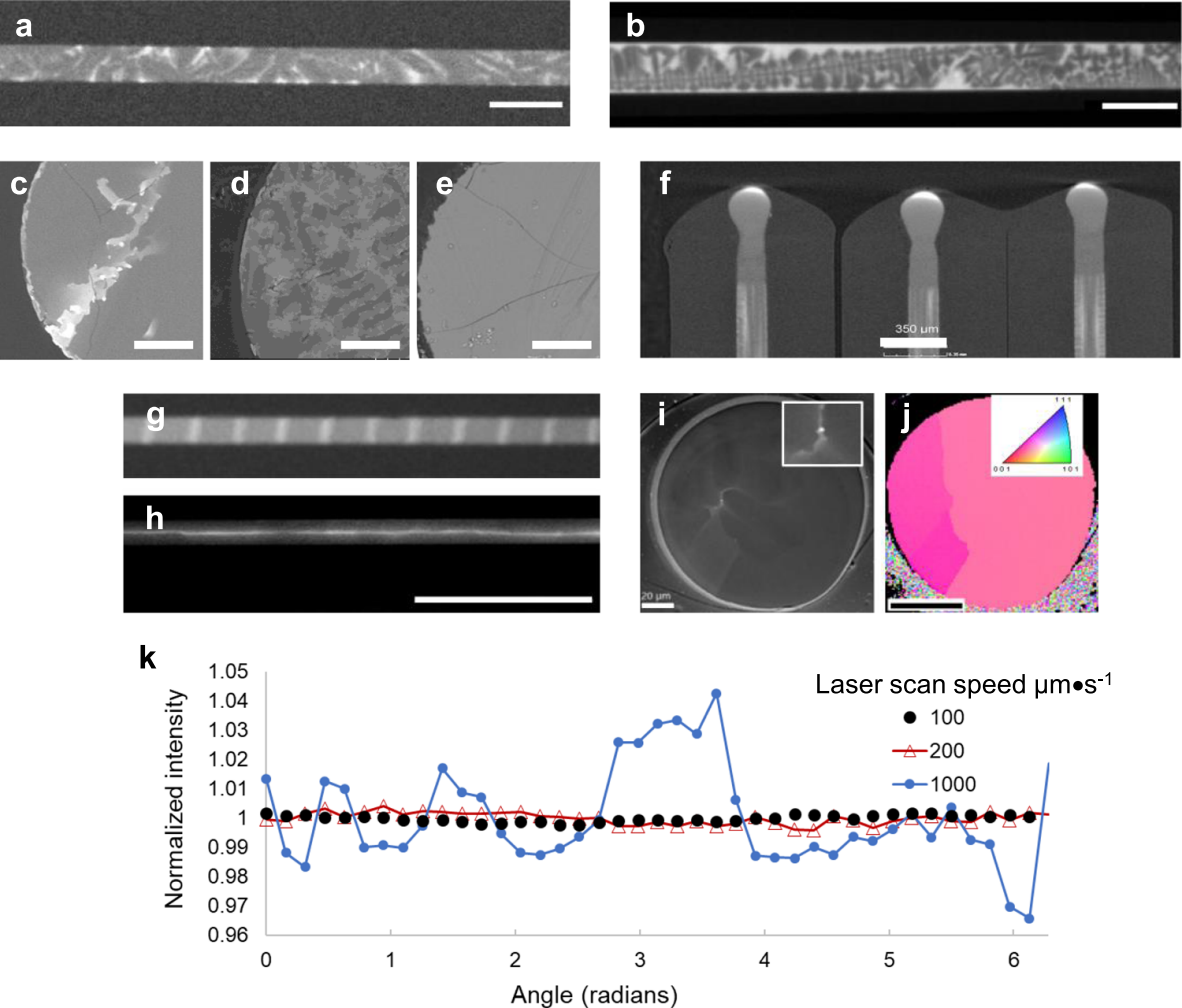
Structures realised in SiGe core fibres. **a** X-ray computed tomography (XCT) of SiGe as-drawn fibres 6% and **b** 20% Ge. Si is a darker material and has crystallised with a dendritic form in the high Ge-concentration sample; grain boundaries are indicated by orientation change. Cross-sectional backscattered electron (BSE) images of **c** as-drawn 6% Ge core and **d** 20% Ge as drawn core, and **e** laser annealed 20% Ge core. Laser-written structures observed by XCT include **f** Ge rich endcaps, **g** gratings and **h** Ge-rich core fibre where **i** BSE image and **j** electron backscatter diffraction indicating high crystalline quality are also shown. Backscattered electron image intensity, an indicator of Ge concentration, **k** as a function of laser scan speed for 6 at% Ge fibres. Scale bar, 200 µm (**a**, **b**), 20 µm (**c**–**e**), 350 µm (**f**), 1 mm (**h**), 20 µm (**i**) and 50 µm (**j**). Images (**a**, **b**, **g**) are (unmodified from ref. ^46^; panels **c**–**e** and **k** modified, are from ref. ^75^. Panels **h**–**j** (modified) are reprinted/adapted with permission from ref. ^120^ © The Optical Society, and **f** (unmodified) is reprinted with permission from ref. ^122^, © The Optical Society.

For the SiGe system, with the phase diagram shown in Fig. 4, fibres typically are drawn at temperatures of ~1950 °C, based on the use of silica as the cladding. SiGe based fibres with Ge concentrations from 1 at%^99^ to 46 at%^118^ have been studied, both as continuous fibre cores^46,72,75,118^, and as source material for particles^48^ within the silica cladding. Compositional variations due to the preferential solidification of silicon from the melt are observed in the as-drawn material^75,118^, as seen in Fig. 5a–d, with a more homogeneous structure obtained after CO_2_ laser annealing^46,99,119,120^, at temperatures estimated to exceed 1800 °C, as shown in Fig. 5e.

The lower melting point of the Ge allows it to act as a solvent, promoting the formation of large single crystalline core regions^121^. The thermal segregation of the elements may be exploited for the fabrication of compositionally structured cores as shown in Fig. 5f-j. Variations in the solidification velocity and imposed thermal gradients have been used to form cores with periodic axial^46^, end-cap^122^ and radial^120^ Ge-rich features, showing the breadth of in-fibre microstructures that can be created in these isomorphic systems. Optical transmission of nominally uniform fibres has not yet been optimised^46,72^, but oven annealing of low Ge concentration HPCVD-fabricated fibres was shown to reduce optical losses^123^, and laser-treated molten core fabricated fibres that were subsequently oven annealed were reported to have decreased losses^119^. This suggests that while slow (~100 µm s^−1^) translation speeds give overall compositional uniformity (Fig. 5k), a combination treatment may be required for the formation of truly homogeneous cores, especially at higher Ge concentrations.

### Binary compounds

For some alloys, thermodynamics favours the formation of compounds with fixed stoichiometries, and one of the highest melting temperature solids has this composition. To date, there are reports of InSb, GaSb and PbTe core glass-clad fibres of this type formed by the molten core method.

Many binary systems have a eutectic point on one side of the desired line compound composition, but choosing compositions on the non-eutectic side (e.g. excess In in the In-Sb system) leaves the overabundant constituent in an elemental metallic form, typically with a low melting point. This leads to the probability that it will act as a solvent, enhancing crystallisation during and/or after drawing. Excess metals can be removed during a directional anneal.

#### InSb

**Fig. 6:**
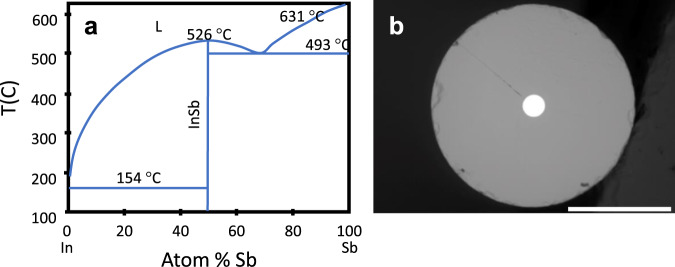
Example of a III-V semiconducting core fibre. **a** Phase diagram for InSb, (L liquid) and **b** SEM cross-section of InSb core fibre. Scale bar, 1 mm. Panel **b** reproduced (unmodified) from ref. ^124^.

InSb core fibres (Fig. 6) in phosphate glass cladding were the first binary semiconductor core fibres to be realised^124^, demonstrating good stoichiometry and structure, although no optical transmission information was reported. Both Raman and X-ray properties of the core were comparable to those of the source (bulk) crystal.

#### GaSb

Subsequently, GaSb core fibres were fabricated by the molten core method^125^ using a commercial borosilicate (Duran^®^) cladding glass, and were CO_2_ laser annealed. This resulted in single crystal core formation and a factor of ten increase in photoluminescence intensity observed at room temperature, the first for direct bandgap semiconductor core fibres. The as-drawn material had excess Sb, which may have acted as a solvent phase during the recrystallisation process, promoting high quality crystal growth.

#### PbTe

**Fig. 7:**
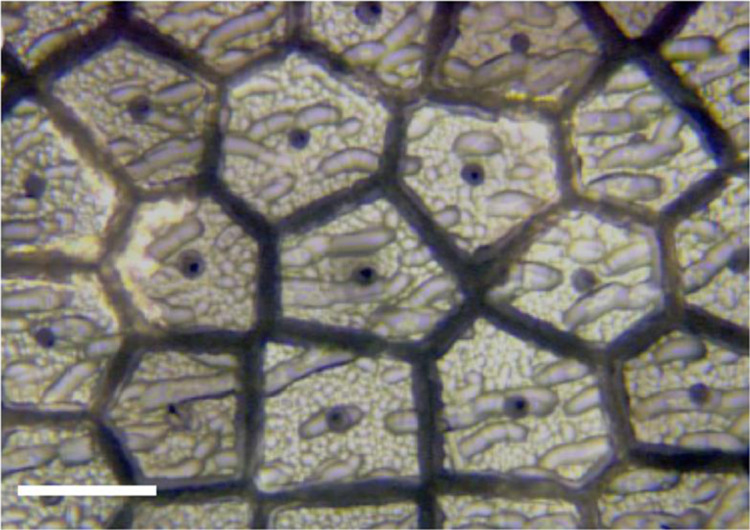
Thermoelectric semiconductor core fibre. Array of PbTe core fibres (modified from ref. ^127^) Scale bar 0.2 mm. Reprinted by permission from Springer Nature, *J. Electron. Mater*. Properties of p- and n-type PbTe Microwires for Thermoelectric Devices, Bhatta, R. P., Henderson, M., Eufrasio, A., Pegg, I. L. & Dutta Copyright 2014.

Lead telluride is a material that came under early study using fibre drawing techniques to fabricate thermoelectric core^126,127^ glass fibres/microwires. Using a borosilicate cladding, good thermoelectric properties and some single crystal cores of less than 10 µm diameter were reported. These were fabricated into bundles, as shown in Fig. 7.

#### ZnSe

Zinc Selenide core fibres^128^, and Cr-doped ZnSe^129^ as well as an array of Ge/ZnSe core fibres have been fabricated by HPCVD^130^. Reactive formation in polymer hosts has also been reported^131^. ZnSe dissociates upon heating, so molten core fabrication is not an appropriate option.

### Two-component eutectic

#### Ge-Sn

**Fig. 8:**
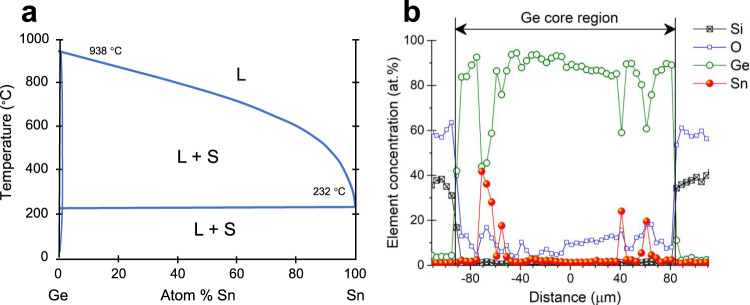
Semiconductor-metal eutectic system. **a** Phase diagram for Ge-Sn, L liquid, S solid, and **b** EDX data showing Sn inclusions. Panel **b** modified from ref. ^132^.

Tin-doped Ge is of interest due to the possibility of forming a direct bandgap Group IV material. Germanium and tin form a eutectic with up to 1% of Sn soluble in Ge as shown in Fig. 8, but less than 0.3% Ge soluble in Sn at equilibrium. Fibres of nominally 9 wt% Ge were drawn by wrapping Ge core with Sn foil^132^ to isolate the core from the borosilicate glass. Optical losses at a wavelength of 3.39 µm were slightly reduced, but the polycrystalline nature of the fibres and residual Sn limited transmission. Post-fabrication treatment of fibres was not reported.

#### Au-Si

**Fig. 9:**
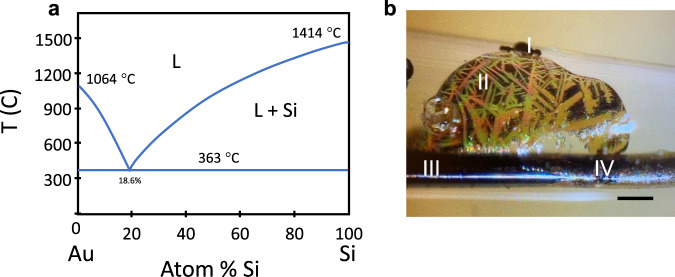
Gold in silicon allows recrystallisation at reduced temperatures. **a** Phase diagram for Au–Si, showing the reduction possible. **b** End point of a gold-removal sweep, where sudden power reduction allowed excess gold to crack the cladding, leaving visible eutectic structure in a thin layer and small metal droplets on the outside of the glass. Features: I: gold droplets, II: eutectic film inside glass crack, III: purified silicon and IV: untreated gold-silicon alloy. Scale bar 0.1 mm.

Silicon and gold form a simple eutectic with very low solid solubility of either component in the other (Fig. 9a). Fibres with silica cladding and nominal 10 at% in silicon concentration were drawn, with core diameters up to 200 µm. Pro-eutectic Au was apparent, but possibly due to the relatively high diffusivity of gold in silicon^133^, no classic eutectic structure was observed. CO_2_ laser refining was used to remove the gold from sections up to 2 cm in length, resulting in formation of either one or two crystalline grains. The gold solvent promoted solidification at a lower temperature than either of the constituents and suppressed nucleation of additional grains. These fibres were shown to have broadband IR and THz transmission^102^, despite residual gold at the 10^16^ cm^−3^ level.

### Complex binary phase diagrams

Thermoelectric core materials in glass-clad fibres have the most complex phase diagrams explored to date.

#### Sn_x_Se_y_

**Fig. 10:**
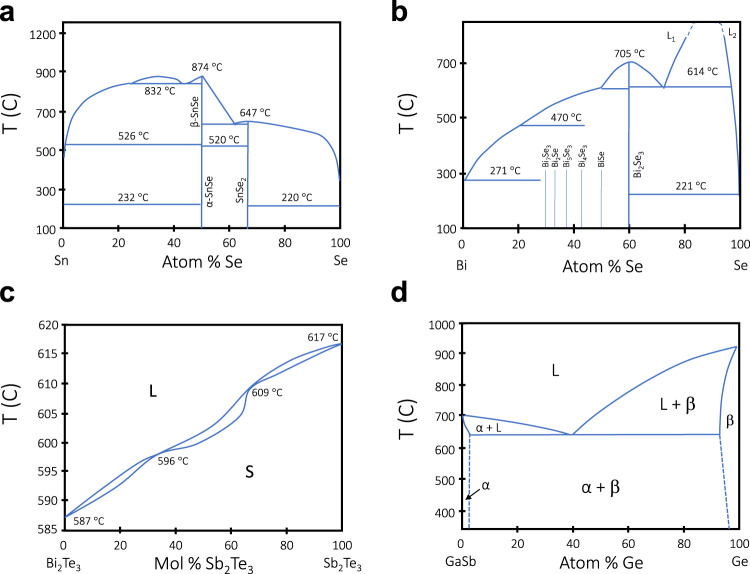
Phase diagrams of more complex systems. **a** Sn–Se, of interest for thermoelectric applications **b** Bi–Se and **c** Bi_2_Te_3_–Sb_2_Te_3,_ also thermoelectrics, and **d** GaSb–Ge, a ternary pseudo-eutectic combination, an analogue of the GaSb–Si system that has been explored. L liquid, S solid, α crystal A with B substitutions; β B with substitutional A.

Tin selenide is a promising material for thermoelectric applications^134^, and was recently drawn in a borosilicate cladding^135^, followed by CO_2_ laser annealing to form single crystalline material^10^. In these experiments, the initial composition of the core was SnSe, and fibres were drawn at 1100 °C. Laser processing was optimal with a speed of 100 µm s^−1^, and temperature gradients of ~2500 K cm^−1^. Despite the complexity of the phase diagram in Fig. 10a, oriented, single crystalline cores with measured lengths of up to 22 mm were realised.

#### Bi_x_Se_y_

Bismuth selenide is another material of interest for thermoelectric applications, and fibres of this material have also been drawn using a borosilicate glass cladding^136,137^. The phase diagram has more than ten intermetallic compounds (Fig. 10b), but both Raman and x-ray analyses confirmed the formation of Bi_2_Se_3_, with thermoelectric figure of merit of the core being slightly higher than that of the starting material^136^.

#### In_x_Se_y_

Similar to the bismuth system, indium–selenium forms many intermetallic phases, and as-drawn fibres using a borosilicate cladding^138^ exhibited a mixture of compositions. Subsequent annealing increased the phase homogeneity of the cores and improved thermoelectric response of the fibres by a factor of two.

### Ternary systems

Addition of a third element to the core can permit reduction of processing temperatures, and/or modification of the electronic band structure of the semiconductor. When the composition of a ternary III–V compound is altered, the bandgap changes smoothly from that of one composition to the other. However, with thin film growth, there is always a constraint due to the need for lattice matching. With fibre draws and the amorphous cladding, this constraint may be reduced. The III–V systems with three components generally have pseudo-binary phase diagrams (two elements from one column are used) with a solid-liquid phase field that can adopt significantly different widths for different materials^139^.

#### Pseudo-binary ternary systems

Bi_x_Sb_(1-x)_Te is reported to be a pseudo-binary system of Bi_2_Te_3_ and Sb_2_Te_3_^140^ (see Fig. 10c). Compounds with additional Te have been shown to have very high thermoelectric figures of merit, in particular Bi_0.5_Sb_1.5_Te_3_, which forms a highly elongated rhombohedral lattice^141^. This core material in borosilicate clad fibres was demonstrated to have as-drawn polycrystalline structure with good thermoelectric properties^137^.

#### In_x_Ga_(1-x)_Sb

Although there is only one preliminary report of a III–V pseudo-binary, In_x_Ga_(1-x)_Sb core fibre^56^, CO_2_ laser processing recrystallised the material successfully, and fabrication of additional materials in this family is worthy of pursuit.

#### Pseudo-eutectic ternary systems

**Fig. 11:**
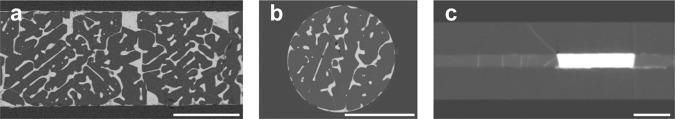
SEM images of the structure of Si/GaSb core in silica clad fibre (bright areas GaSb). The lower melting point of the GaSb allows it to act as a solvent during solidification; the cores are highly crystalline, as evidenced by the segregation to preferential planes in the silicon. **a** longitudinal view, **b** cross-section, **c** segregation of GaSb after annealing. Scale bar, 100 µm (**a**, **b**) and 300 µm (**c**). (ref. ^12^).

There is one ternary pseudo-eutectic that has been reported as a core material in glass-clad fibres: Si/GaSb, where there is little mixing of the III–V components and the silicon. While there is no published phase diagram for this combination, it should be analogous to the diagram for GaSb–Ge, shown in Fig. 10d. The Si/GaSb fibres show classic eutectic structure features, seen in Fig. 11. In fibres of Si with 10 at% GaSb added to the core, the GaSb acted as a low-temperature solvent for the silicon. CO_2_ laser annealing segregated the III–V component, which exhibited slightly broadened photoluminescence at room temperature^12^. The residual Si core was single-crystalline.

## Cladding Materials

While the focus of this review is on the materials and phase equilibria of the semiconducting fibre core phases, it is worthwhile to briefly discuss the cladding glasses. For HPCVD fabrication, the capillary holes of the cladding glass provide a scaffold into which the semiconductor is deposited. For molten core fabrication, the cladding glass is both a crucible for melting of the semiconductor phase and also a carrier of the melt as the fibre is drawn from the molten core preform. In both cases, the glass serves as an optical waveguide cladding for the resultant fibre.

To first order, the cladding glass, usually in the form of a tube (molten core) or a capillary fibre (HPCVD) is selected based on commercial availability and whether it possesses the requisite thermomechanical properties for the system. For example, for molten core fibres, the cladding glass needs to have a draw temperature that exceeds the melting point of the core phase. Secondary considerations include matched core/clad thermal expansion coefficients to minimise residual stress in the fibre. To-date, cladding glasses have included pure silica^68^, borosilicate^83,114^ and phosphate compositions^124^, depending on the core phase employed.

Although commercial availability of the cladding glass makes for more straightforward studies, there are limitations in the range of compositions, hence properties, that are possible. Several reports have identified ‘designer' cladding glasses for core-specific fibres^142–144^, and definitely represent an opportunity for continued future developments.

## Emerging Areas

The predominant application of semiconductor core fibres to date has been in optics, as emphasised by the large number of publications in that area, addressing improved broadband transmission^102^, photodetection^70^ and light sources^93,106,125^. As control over core composition and structure have improved, additional glass development and fibre applications are on the horizon.

### Photonics

Both linear and non-linear optical applications will expand as the properties of the fibres continue to improve. Radial composition gradients in crystalline semiconductor cores allow for lower losses in the IR, where presently the glass cladding is a limitation, and reduction of cladding thickness to provide flexibility for long wavelength applications will permit industrial use of lasers that suffer from atmospheric absorption. In addition to the IR applications that can be explored with the traditional semiconductors, one of the areas that is of great interest is the development of crystalline oxide core^145,146^ materials. This would allow further exploitation of both second and third order susceptibilities in the fibre geometry, for traditional non-linear optics as well as entanglement and other quantum phenomena.

The optical quality of silicon core fibres is now competitive with silicon-on-insulator technology, and the advantages of long interaction lengths, and combined electrical/optical control will potentially make this rapid/environmentally low-impact technology a competitive source of small-dimensional semiconductor devices. Transferring the advances in materials processing from fibres to two dimensional (2D) platforms is an exciting possibility, with recent demonstration of semiconductor waveguide fabrication leading the way^147^. Although there has been dramatic progress in light source integration in the last years^148–150^, the continued parallel development of different approaches suggests that there is not yet a clear optimum solution. Integration of the light source into fibres, particularly for the processor–network interface would be a step forward and could simplify chip processing. Progress with fibre nanospike couplers will support this transition once robust fibre-based sources are developed.

Additional progress on integration of fibres or laser processing into 2D circuits are on the horizon. In parallel, the demonstration of high-quality fibre product will attract interest from other optical sub-fields, and broaden the spectrum of applications, such as optical computing.

### Optoelectronics

Molten core fibres have been explored for fabrication of p-n diodes^151^ and solar cells^76,152^. Applications based on the electrical properties are increasing, particularly for sensors^153–155^. Stress effects have been shown to alter the bandgap of silicon^156^, and it may be possible to produce direct bandgap GeSn with reduced Sn content^157^ when fibre stress effects are harnessed. Further development of III–V materials is anticipated. There are preliminary reports of diode formation due to spatial segregation of the elements in a fibre^158^, and in-line fabricated devices have already been realised in polymer based fibres^1^. Using fibre bundles as pixelated sensors is an attractive proposition. Electrode fabrication is under study^159^, and further progress in this area will advance the field significantly.

### Thermoelectrics

The recent increase in the number of papers and research groups fabricating thermoelectric fibres^126,127,136–138^ is evidence of improved crystal composition and structural control, as well as the recognition of the fibre drawing technique as a valuable method for achieving the geometry required for affordable assembly of devices. The resulting fibres can be woven into fabrics to generate electricity from the temperature difference between skin and the environment, or to provide cooling with modest energy input. Incorporation of these fibres in surfaces proximal to heat engines would allow capture of some of the waste thermal energy. Assembly of these fibres into arrays is a natural use of the geometry.

### Gravity wave interferometers

One type of sensor expected to benefit from advances in silicon core fibre fabrication developments is the gravity wave detector, where the next generation interferometers anticipate use of single crystal suspension systems^11^. Non-destructive techniques for assessing the grain structure of fibres using conventional x-ray diffraction methods were recently introduced in the Supplementary Material of the paper by Song, et al^12^, and will be of use in this and other applications where structure is a primary concern.

### Other applications

Some of the materials of interest for spintronics are also candidates for fabrication using the molten core fibre route. Initial experiments will continue with thin film samples, but it is possible that the long-term development of these materials will benefit from the simplicity of the fibre draw method. In a broader context, materials that will benefit from periodic polarisation or property variations will benefit from the fibre geometry; for example applied magnetic fields could be varied during travelling melt zone treatment, or chiral structures could be inscribed by rotating the fibre during annealing. While we have emphasised the phase diagrams as a basis for interpreting the core behaviour, it is also worth considering whether the fibres represent a materials-efficient method for studying phase diagrams, particularly in metastable systems.

## Outlook

Key themes that emerge from a survey of the literature in the context of the materials science of semiconductor core fibres are the exciting advances in the growth of single crystal cores and developments in compositional structuring. Consideration of phase diagrams, coupled with analysis of the kinetics inherent during fibre fabrication and post-process treatment is opening doors to major performance improvements. There are numerous areas where additional studies are needed, particularly the role of cladding-induced stress, determination of whether clean grain boundaries are deleterious for optical applications and the best methods to limit the inclusion of, or to remove impurities from fabricated fibre.

While this article emphasises the use of phase information to assist in the formation of desired materials, the fibre format for fundamental studies of phase diagrams and thermal treatments is also an area worthy of investigation—the small amounts of materials needed, the range of thermal treatments that can be applied and the characterisation accessibility of the core combine to make this a powerful approach for future studies^120,122,124^.
